# *PLK1* inhibitor facilitates the suppressing effect of temozolomide on human brain glioma stem cells

**DOI:** 10.1111/jcmm.13793

**Published:** 2018-08-22

**Authors:** Naijie Liu, Guozhang Hu, Han Wang, Zhaohui Li, Zhigang Guo

**Affiliations:** ^1^ Department of Neurosurgery China‐Japan Union Hospital of Jilin University Changchun China; ^2^ Department of First‐aid Medicine China‐Japan Union Hospital of Jilin University Changchun China; ^3^ Department of Clinical Laboratory Changchun Chinese Medicine University Affiliated Hospital Changchun China

**Keywords:** BI2536, glioma stem cells, *PLK1*, temozolomide

## Abstract

Glioblastoma is the most frequent and most aggressive brain tumour in adults. Temozolomide is an oral chemotherapy drug and one of the major components of chemotherapy regimens used as a treatment of some brain cancers. We examined the tolerance of stem cells isolated from glioma cell line U87 and U251 to temozolomide (TMZ) and explored the effect of PLK1 (Polo like kinase 1) protein expression on TMZ sensibility. In our results, the inhibitory effects of TMZ on glioma cells U87, U251 and its stem cells were confirmed to be dose dependent and time dependent. Compared with glioma cells, the glioma stem cells showed a greater degree of tolerance. As the concentration of TMZ increased, the expression of PLK1 protein increased in U87 cells, CD133^+^ U87 stem cells and CD133‐ U87 cells. The increase range of PLK1 protein was large in CD133^+^ U87 stem cells and small in CD133‐ U87 cells. TMZ treatment in cells with low PLK1 protein expression efficiently suppressed the cell proliferation and sphere formation, while G2/M arrest was strongly induced. What's more, TMZ and PLK1 inhibitor synergize to inhibit glioma growth in vivo. In conclusion, our results suggest that down‐regulation of PLK1 protein enhanced the inhibition of TMZ on glioma stem cells, suggesting its clinical value to adverse TMZ resistance in glioma treatment.

## 1 INTRODUCTION

Glioblastoma has been classified as the highest grade glioma (grade IV) by the WHO.^1^ Glioblastoma is the most irresistible malignant brain cancer in adults.^2^ The recurrence rate is very high with a median survival of 14.6 months despite aggressive multimodal therapy.^3^ Glioblastoma were identified as a kind of solid cancers in which tumour cells with stem cell‐like features, so‐called cancer stem cells (CSC).^4^ Cancer stem cells are thought to be the product by asymmetric division of oligodendrocyte progenitor cells (OPCs) or neural stem cells (NSCs), sharing same markers including SOX2, PDGFRβ, CD133, Nestin, and others.^5^ Glioma stem cells are a group of self‐renewing and tumorigenic subpopulation of glioma cells, an important factor of relapse and chemoresistance in glioblastoma.^6^ Glioma stem cells are responsible for cancer initiation, maintenance, progression and recurrence.^7^ However, the role of cancer stem cells in glioblastoma is still unclear.

Temozolomide (TMZ) is a DNA‐alkylating agent that causes lethal DNA lesions in fast‐dividing cancer cells.^8^ TMZ is widely used to treat primary or metastatic brain cancer, which had efficacy on the recovery of patients.^9^ It was reported that BIS or HSF1 knockdown combined with TMZ treatment increased tumour apoptosis while cancer stem‐like properties was suppressed, such as SOX2 protein expression.^10^ Hao et al demonstrated that resveratrol combination with TMZ had significant efficacy on glioblastoma progression.^11^ Some studies also explored the effects of TMZ on glioblastoma cells activities. For example, miR‐146b‐5p enhanced cell duplication and impeded cell apoptosis through mediating TMZ resistance in glioblastoma cells.^12^ The mechanism of TMZ interacting with other molecules in glioma cells is crucial to the therapies of glioma.

Polo‐like kinase 1 protein (*PLK1*) was reported to highly express in various tumours. By overriding the G2‐M DNA damage and spindle checkpoints, overexpression of *PLK1* can promote chromosome aneuploidy and instability.^13^ Chemical inhibitors or knockdown of *PLK1* decreased medulloblastoma cells growth.^13^ Robin et al illuminated that *PLK1* was promoted in CD133‐positive cells and combined inhibition of *PLK1* and BRAF resulted in significantly greater pro‐apoptotica and anti‐proliferative effects than those achieved by monotherapy.^5^ Koncar et al researched the interaction of TMZ and *PLK1* in glioma, and reported that combination treatment of TMZ and a *PLK1* inhibitor BI2536 caused significant cancer shrinkage and tumour regression in in vivo experiments, while TMZ or BI2536 alone had little effect on tumour growth.^14^ The influence of TMZ and *PLK1* on glioma cellular activities needs to be further studied.

In this study, we evaluated the effects of *PLK1* on glioblastoma and the synergistic inhibition effect of *PLK1* inhibitor combined with TMZ on human brain glioma stem cells in vitro and vivo. Our study suggested that *PLK1* inhibitors may be a novel therapies target for glioma treatment.

## 2 MATERIALS AND METHODS

### 2.1 U87 and U251 CD133‐positive cells isolation and culture

The human glioblastoma cell line U87 and U251 was obtained commercially from ATCC and were cultured in Dulbecco's modified Eagle's medium (DMEM, Invitrogen, Carlsbad, CA) supplemented with 10% bovine serum and 100 μg/mL streptomycin. For the isolation, U87 and U251 cells were suspended at FcR reagents were added for blocking. Microbeads cultured with CD133 antibody (ab19892, Abcam, Cambridge, MA) were then added, and the mixture was cultured at 37°C for 1 hour. Cells collected was recognized as CD133‐ fractions while cells obtained after removing the magnetic holder was diagnosed as CD133^+^ cells, also as glioma stem cells. Glioma stem cells were cultured in a serum‐free DMEM‐F12 medium (Invitrogen) supplemented with 10 ng/mL basic fibroblast growth factor (bFGF, Invitrogen), 20 mg/mL epidermal growth factor (EGF, Invitrogen) and 2% B27 (Invitrogen) under 5% CO_2_ at 37°C.

### 2.2 Cell transfection

CD133^+^ U87 stem cells and CD133^+^ U251 stem cells were assigned to Blank group, control group, *PLK1* inhibitor BI2536 group (treated with 0.5 nmol/L BI2536, Selleck Chemicals, Houston, TX), *PLK1* inhibitor Volasertib group (treated with 0.5 nmol/L Volasertib, Selleck Chemicals), pcDNA3.1 group, pcDNA3.1‐*PLK1* group (cells transfected with *PLK1*‐pcDNA3.1), si‐NC group, *PLK1‐*siRNA1 group (cells transfected with *PLK1*‐specific siRNA1) and *PLK1‐*siRNA2 group (cells transfected with *PLK1*‐specific siRNA2). The sequence of *PLK1* siRNA is listed in Table 1. The oligonucleotides were purchased from Gene PharmaCo., Ltd. (Shanghai, China). U87 and U251 stem cells were plated in antibiotic‐free medium. Then, the medium was changed to serum‐free Opti‐MEM. Transfection was performed under the guidelines of Lipofectamine 2000 (Invitrogen Inc.).

**Table 1:** siRNA sequence of *PLK1*

Gene	Sequence
siRNA1	5′‐GCTGCACAAGAGGAGGAAA‐3′
siNC‐1	5′‐GCTAACAGGAGGGACGAAA‐3′
siRNA2	5′‐GCACCGAAACCGAGTTATT‐3′
siNC‐2	5′‐GCAGAAACCGAGTTCCATT‐3′

### 2.3 Cell viability assay

The human brain glioma U87 cells, U251 cells and stem cells were correspondingly seeded in 10% FBS cell culture medium and stem cell culture medium. The culture medium was moved and DMEM‐F12 (Hyclone, Logan, UT, USA) was added. TMZ (human glioma U87, CD133‐ U87, U251 and CD133‐ U251 cells: serum medium + TMZ, human glioma U87 and U251 stem cells: serum‐free medium + TMZ) were added to 96‐well plates at stated concentration. Cells were collected at incubation for 24, 48 and 72 hours and then treated with CCK‐8 (10 μL/well, Beyotime, Shanghai, China) reagent for another 5 hours. The absorbance at 450 nm was measured by an automatic enzyme‐linked immune detector (Multiskan MK3, Thermo Labsystems, Helsinki, Finland).

### 2.4 Flow cytometry assay

Monolayer cultured human glioma CD133‐ U87 and CD133‐ U251 cells and stem cells were fixed in the ice‐cold ethanol (70%) at −20°C overnight. Then the cells were first incubated in the RNAse and stained with propidium iodide. Cell cycle analysis was carried out on a FACS Calibur flow cytometer (Thermo Fisher scientific, Inc., Beijing, China), and the obtained data were analysed using Cell Quest software (BD Biosciences, San Jose, CA, USA).

### 2.5 QRT‐PCR

Total RNA was isolated using Trizol Reagent (Invitrogen) and reverse‐transcribed using the High Capacity kit (Applied Biosystems, Foster City, CA). The qRT‐PCR procedures were operated according to the Maxima SYBR Green qPCR Master Mix (2X) kit (Thermo) protocol. Relative gene expression was analysed by 2^−▵▵Ct^ method. The RNA primers used are listed in Table 2.

**Table 2:** Prime sequences for qRT‐PCR

Gene	Prime sequence
PLK1	F: 5′‐ACCCAGGAGAGAGGTCCAGT 3′
	R: 5′‐CACGGCACAAAGACGATG‐3′
GADPH	F: 5′‐AACGGATTTGGTCGTATTG‐3′
	R: 5′‐GGAAGATGGTGATGGGATT‐3′

### 2.6 Western blot

Cells were washed in ice‐cold PBS and then treated with a RIPA protein lysis buffer (Beyotime) to prepare protein lysates. The total protein of tumour tissues also obtained with a RIPA protein lysis buffer. The protein concentrations in the cell lysates were measured by BCA protein assay kits (Pierce, Rockford, IL) and then calibrated by standard bovine serum albumin concentrations. Total proteins for each cell lysate sample were separated by SDS‐PAGE and transferred to PVDF membranes (Bio‐ Rad, Richmond, CA, USA). Five % bovine serum albumin in Tris buffer (TBS) blocked the membrane overnight at 4°C. Primary antibodies specific to target proteins (Anti‐*PLK1*, ab17056, 1 μg/mL; anti‐GAPDH, ab181603, 1:10 000, Abcam) were used for probing, and corresponding HRP‐labelled goat anti‐rabbit (IgG‐HRP, ab6721,1: 1000, Abcam) were used for detection. The enhanced chemiluminescence (ECL) Detection System (Thermo Scientific, Rockford, IL) was used to visualize the immunoreactive proteins, which were then photographed and observed under a microscope (Bio‐Rad). GAPDH was considered as the internal reference.

### 2.7 Soft agar colony formation assay

The bottom layer of soft agar (0.9%) was prepared in a six‐well plate, and the top layer (0.5%) was prepared with 5 × 10^4^/mL CD133^+^ stem cells, CD133‐ glioma cells and U87 or U251 cells in single‐cell suspension. The cells were exposed to 40 μmol/L TMZ for 2 weeks and cultured in an incubator at 37°C with 5% CO_2_. Colony formation was observed by microscopy, and colonies of >30 cells were counted under a microscope. The experiments were repeated in triplicate.

### 2.8 GSC sphere‐forming assays

GSC spheres were enzymatically dissociated to single cells and re‐plated in 96‐well plates at optimal density (500‐1000 cells) in non‐adherent conditions. Cells were cultured in serum‐free DMEM‐F12 medium (Invitrogen) supplemented with 10 ng/mL bFGF, 20 ng/mL EGF and 2% B27. Half of the medium was renewed every other day, and after 6 days, the cells were fixed with 4% formalin. The cells were photographed and spheres larger than 50 μm were counted.

### 2.9 Tumour xenografts in mice

Forty‐two male athymic BALB/c nude mice (4‐week‐old) were obtained from the Shanghai Medical Experimental Animal Care Commission (Shanghai, China). All animal procedures and experimental protocols were approved by Laboratory Animal Ethics Committee of China‐Japan Union Hospital of Jilin University.

To establish xenograft tumours, CD133^+^ U87 stem cells (8 × 10^6^ in 200 μL of medium) were injected subcutaneously into the dorsal flank of each mouse. Once the tumour volume reached 200 mm^3^, animals were randomized into seven groups: Blank, control, TMZ, BI2536, Volasertib, TMZ+BI2536, TMZ+Volasertib (n = 6 mice per cohort). TMZ (25 mg/kg formulated in 10% DMSO in sterile PBS) was delivered via oral gavage every 2 days (six doses in total). BI‐2536 was dissolved in 10% DMSO in sterile PBS at 20 mg/kg concentration and delivered intravenously under a twice per week schedule (six doses in total). Volasertib was dissolved in 10% DMSO in sterile PBS at 10 mg/kg concentration and delivered intravenously under a twice per week schedule (six doses in total). Each mouse's tumour was measured every 3 days after the first injection, by a Vernier calliper along two perpendicular axes. The volume of the tumour was calculated with the formula: volume = (length × width^2^)/2. Thirty days after the injection, the mice were killed and the tumours were dissected for analyses.

### 2.10 Haematoxylin and eosin (HE) staining

The tumour tissues obtained from mice were fixed with 10% formaldehyde, embedded in paraffin, cut into 4 μm sections and stained with haematoxylin and eosin (HE). Steps for HE staining were as follows: the tissues were sliced up, and then, the sections were baked at 70°C for 4 hours, dewaxed, hydrated in distilled water, stained with haematoxylin (1 minute), differentiated in hydrochloric acid alcohol, blued in ammonia water, counterstained with eosin (7 seconds), dehydrated with ethanol at different concentrations (75%, 90% and anhydrous ethanol), transparentized with xylene I and xylene II, and finally mounted in neutral gum. The tumour tissue sections were observed under a microscope.

### 2.11 Statistical analysis

Each assay was conducted in triplicate. Statistical analysis was performed using GraphPad Prism 6.0 software (GraphPad Software, La Jolla, CA). Student's *t* test was applied to compare the differences between two groups, while the differences between multi‐samples were analysed by analysis of variance (ANOVA). *P* value of <0.05 was considered statistically significant.

## 3 RESULTS

### 3.1 TMZ suppressed the cell viability and induced cell cycle arrest of glioma cells and glioma stem cells

**Figure 1:**
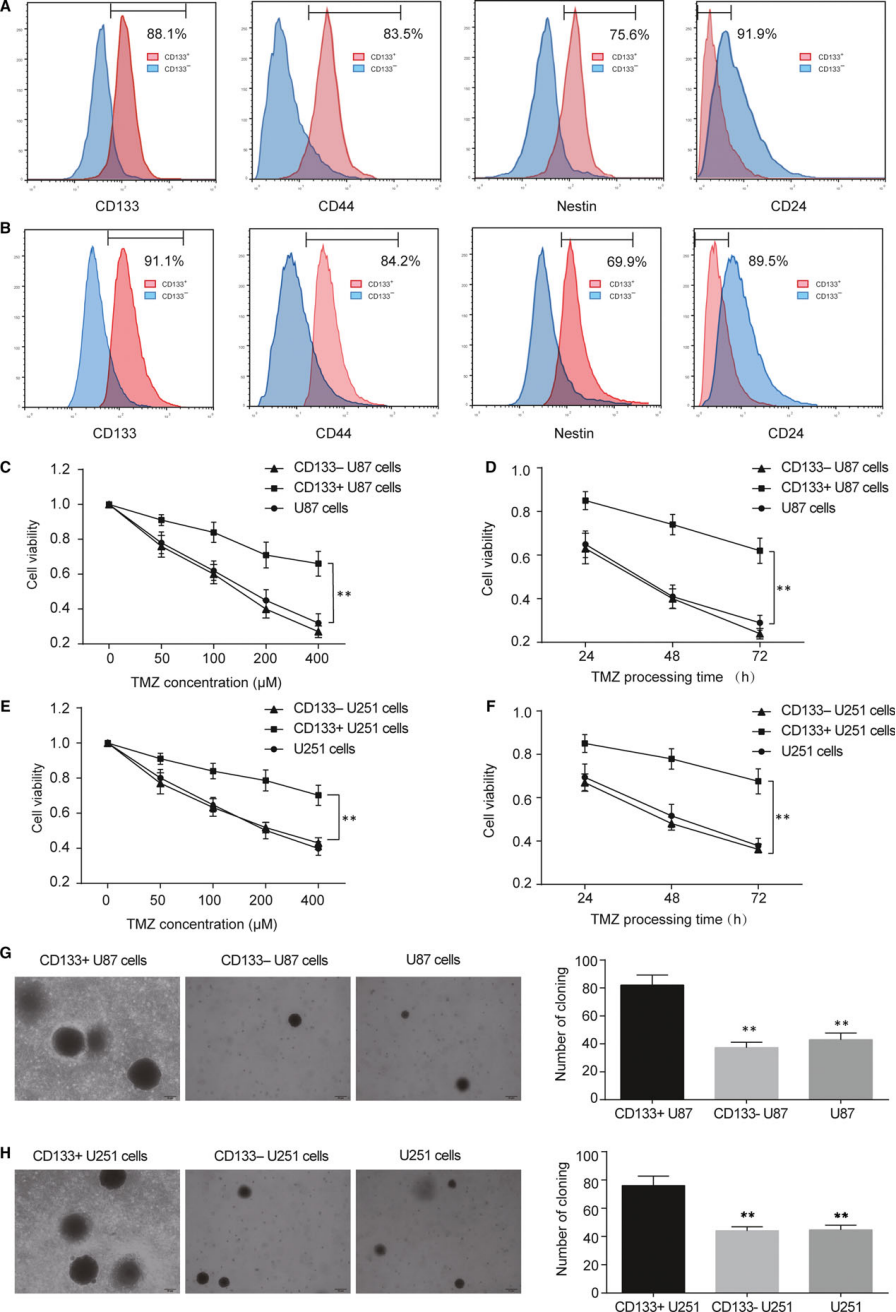
U87 CD133+ cells and U251 CD133+ cells were more resistant to TMZ. A. Sorted by immunomagnetic beads, protein expression of CD133+ and CD133‐ U87 cells were determined with flow cytometry. Proportions of CD133+, CD44+, Nestin+ or CD24‐ cells were lined out, supporting that CD133+ U87 cells were glioma stem cells. B. The isolation and identification of CD133+ U251 glioma stem cells. C. Treated with different concentration of TMZ for 24 hours, cell viabilities of U87, CD133+ U87 and CD133‐ U87 cells were determined. The inhibition was dose‐dependent and U87 cells are more sensitive to TMZ compared with CD133+ U87 cells. C. Treated by 100 μM TMZ, U87, CD133+ U87 and CD133‐ U87 cells were collected at 24 h, 48 h and 72 h respectively. The inhibition was confirmed to be time ‐dependent and viability of CD133+ U87 cells reduced less than U87 cells. D. U251, CD133+ U251 and CD133‐ U251 cells were treated with different concentration of TMZ for 24 hours and then cell viabilities were measured by CCK‐8 assay. CD133+ U251 cells were more resistant to TMZ compared with CD133‐ U87 cells or U87 cells. E. Cell viability of U251, CD133+ U251 and CD133‐ U251 cells with 100 μM TMZ treatment for 24 h, 48 h and 72 h respectively. F. Clonogenesis ability of the U87, CD133+ U87 and CD133‐ U87 cells were tested by soft agarose assay after 40 μM TMZ treatments for 2 weeks. G. Cloning efficiency was measured by counting clone number growing in soft agar in U87, CD133+ U87 and CD133‐ U87 cells after exposing to 40 ?M TMZ for 2 weeks. H. Cloning efficiency of U251, CD133+ U251 and CD133‐ U251 cells after exposing to 40 μM TMZ for 2 weeks. Data was presented as Mean ± SD (standard deviation) from triple experiments. **P < 0.01 compared with CD133+ U87 cells or CD133+ U251 cells

CD133‐positive glioma stem cells were isolated from glioma cells U87 and U251 by CD133 antibody beads. The results revealed that CD133^+^ cell fraction accounted for 1.46% of the total population in U87 cells. The corresponding stem cell‐specific cell surface antigens were labelled with antibodies of CD133, CD44, Nestin and CD24, respectively. The expression of CD133, CD44, Nestin and CD24 in CD133‐positive and CD133‐negative cells after U87 separation were compared. In CD133‐positive U87 cells, the positive rate of CD133 88.1%, CD44 positive cells accounted for 83.5%, Nestin positive cells accounted for 75.9%, while CD24 was mainly negative, CD24 negative cells accounted for 91.9% (Figure 1A). According to these data, the sorted U87 cells were mainly glioma stem cells. In the same way, we get U251 stem cells with 84.2% CD44‐positive cells, 69.9% Nestin‐positive cells and 89.5% CD24‐negative cells (Figure 1B). U87 cells, CD133^+^ U87 cells and CD133‐ U87 cells were cultured in the corresponding medium with different concentrations of TMZ. Cells were collected 24 hours later, and cell viability was measured by CCK‐8 method. The inhibitory effect of TMZ on U87 cells, CD133^+^ U87 cells and CD133‐ U87 cells was positively correlated with the concentration of TMZ. CD133^+^ U87 cells exhibited stronger cell viability compared with U87 cells, showing strong tolerance (Figure 1C, *P* < 0.01). However, there was no significant difference between CD133‐ U87 cells and U87 cells. The results of CCK‐8 showed that the inhibitory effect of TMZ on cells increased with time. The inhibitory effect on CD133^+^ U87 cells was significantly weaker than that on U87 cells, but the inhibitory effect on U87 CD133‐ cells was no significant difference with that on U87 cells (Figure 1D, *P* < 0.01). Soft agar assay suggested that the clone size and quantity in CD133^+^ U87 cells were more than CD133‐ U87 cells and U87 cells after exposing to 40 μmol/L TMZ for 2 weeks (Figure 1G, *P* < 0.01). Similar results were observed in U251 cells, CD133^+^ U251 cells and CD133‐ U251 cells (Figure 1E,F,H, *P* < 0.01). These results indicated that glioma stem cells were more tolerant to TMZ than glioma cells.

**Figure 2:**
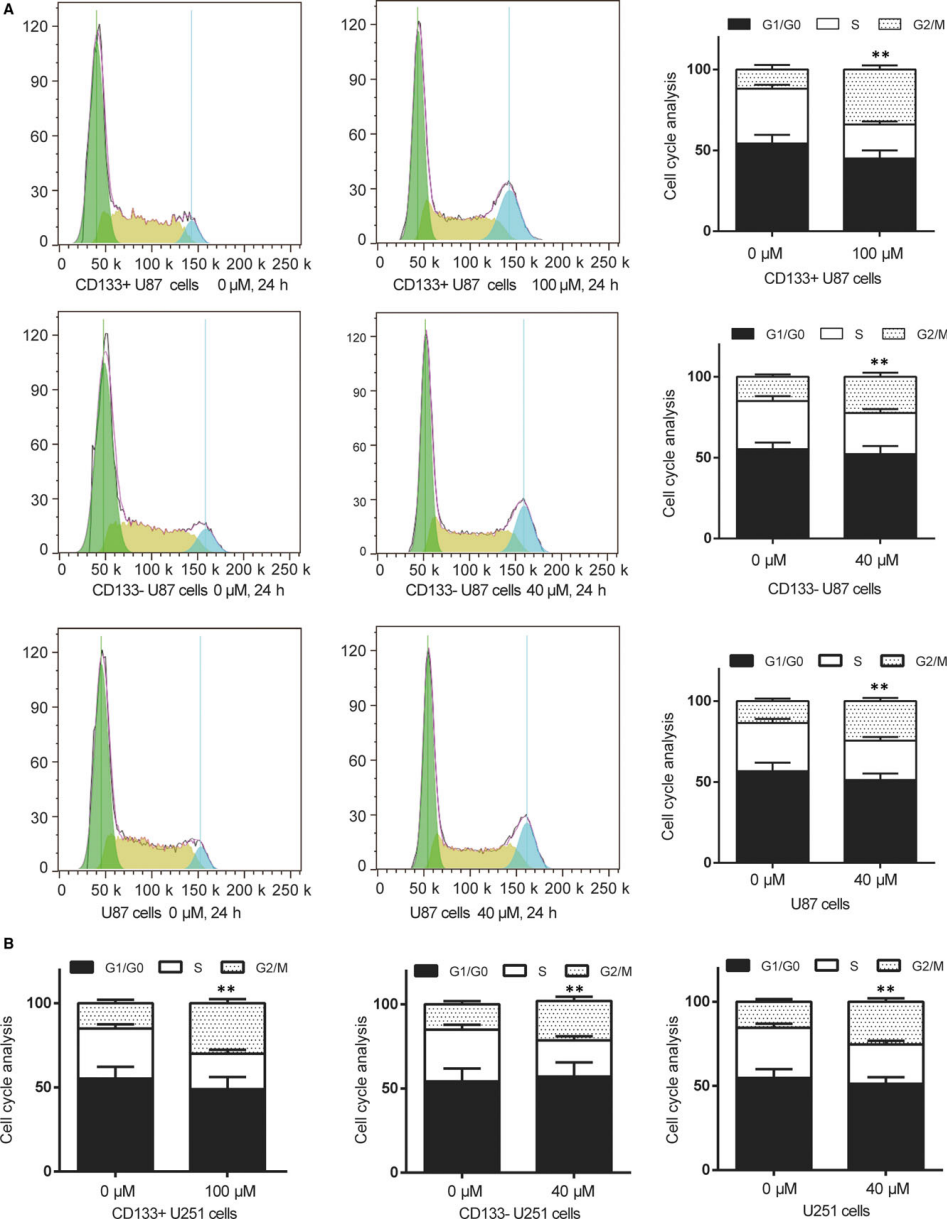
TMZ Induced Cell Cycle Arrest of U87 CD133^+^ Cells and U251 CD133^+^ Cells. A, After treated by 100 μmol/L TMZ to U87 CD133^+^ cells, 40 μmol/L TMZ to U87 CD133‐ and U87 cells for 24 h, cells were collected and cell cycle was analysed using PI staining and flow cytometry. B, After treated by 100 μmol/L TMZ to U251 CD133^+^ cells, 40 μmol/L TMZ to U251 CD133‐ and U251 cells for 48 h, cells were collected and cell cycle was analysed using PI staining and flow cytometry. Data were presented as Mean ± SD (standard deviation) from triple experiments. **P < 0.01 compared with 0 μmol/L TMZ groups

We further examined the effect of TMZ on the cell cycle (Figure 2A, *P* < 0.01) and found that U87 cells, CD133^+^ U87 cells and CD133‐ U87 cells had a certain degree of G2/M block after 48 hours of TMZ treatment. Cells were arrested in G2/M period, and the arrest was stronger in U87 cells compared with CD133^+^ U87 cells. Besides, TMZ could also induce U251 cells, CD133^+^ U251 cells and CD133‐ U251 cells G2/M arrest and the effect in CD133^+^ U251 cells was stronger than CD133‐ U251 cells and U251 cells (Figure 2B, *P* < 0.01).

### 3.2 PLK1 was involved in TMZ tolerance

**Figure 3:**
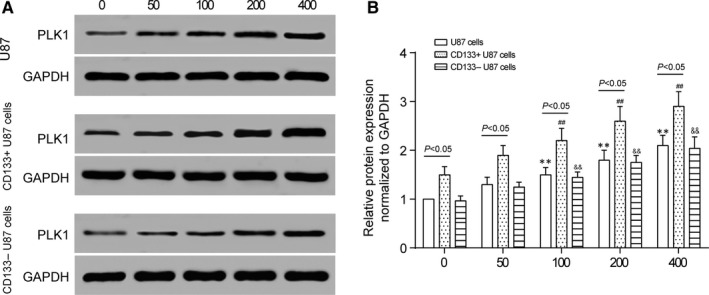
PLK1 Expression was Probably Involved in TMZ Sensibility. A, After treated by different concentration of TMZ for 24 h, U87 cells, CD133^+^ U87 cells and CD133‐ U87 cells were harvested. Protein level of PLK1 was determined by Western blot. B, Analysed by ImageJ, PLK1 expression was quantified and shown in the histogram. Data were presented as Mean ± SD (standard deviation) from triple experiments. **P < 0.01 compared with untreated U87 cells. ^##^ *P* < 0.01 compared with untreated CD133^+^ U87 cells. ^&&^ *P* < 0.01 compared with untreated CD133‐ U87 cells

We performed Western blot assay to detect the *PLK1* expression with the participation of TMZ. The results exhibited increase in PLK1 protein expression in U87 cells, CD133^+^ U87 cells and CD133‐ U87 cells with the increase in TMZ concentration (Figure 3, *P* < 0.01). This result indicated that TMZ treatment activated PLK1‐related signalling pathways and PLK1 may be involved in TMZ tolerance. Additionally, PLK1 protein expression in CD133^+^ U87 cells was higher than that in U87 cells and CD133‐ U87 cells with or without TMZ treatment which indicated that TMZ tolerance may mainly be mediated by CD133^+^ U87 cells.

### 3.3 The effect of PLK1 on tolerance of CD133^+^ U87 stem cells and CD133^+^ U251 stem cells

**Figure 4:**
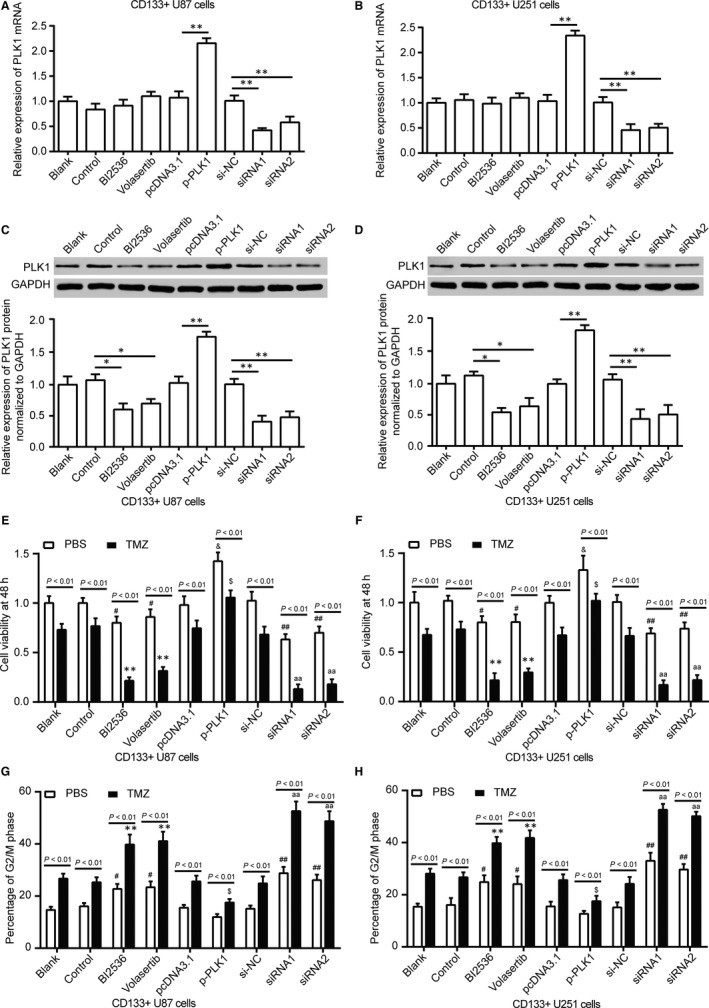
The Effect of PLK1 on Tolerance of CD133^+^ U87 Stem Cells. A‐B, CD133^+^ U87 and CD133^+^ U251 stem cells were divided into blank group, control group, *PLK1* inhibitor BI2536 group (cells treated with 0.5 nmol/L *PLK1* inhibitor BI2536), *PLK1* inhibitor Volasertib group (cells treated with 0.5 nmol/L *PLK1* inhibitor Volasertib), pcDNA3.1 group, pcDNA3.1‐*PLK1* group (cells transfected with pcDNA3.1‐*PLK1*), si‐NC group, *PLK1*‐siRNA1 group (cells transfected with *PLK1*‐specific siRNA1) and *PLK1*‐siRNA2 group (cells transfected with *PLK1*‐specific siRNA2). The total RNA was isolated and the expression of PLK1 mRNA was analysed by RT‐qPCR. **P < 0.01. C‐D, The total protein was analysed, and PLK1 protein expression was determined by Western blot in CD133^+^ U87 and CD133^+^ U251 stem cells. *P < 0.05, **P < 0.01. E‐F, Treated with PBS or 100 μmol/L TMZ for 24 h, cell viability of CD133^+^ U87 and CD133^+^ U251 stem cells was detected using CCK‐8 assay. TMZ efficiently suppressed the cell viability of CD133^+^ U87 stem cells with low PLK1 protein level. G‐H, PI staining and flow cytometry was performed to analyse the cell cycle of CD133^+^ U87 and CD133^+^ U251 stem cells in nine groups. TMZ induced stronger G2/M arrest in CD133^+^ U87 and CD133^+^ U251 stem cells with low PLK1 protein level. ^#^ *P* < 0.05 compared with control group which cells treated with PBS. **P < 0.01 compared with control group which cells treated with 100 μmol/L TMZ. ^&^ *P* < 0.05 compared with pcDNA3.1 group which cells treated with PBS. ^$^ *P* < 0.05 compared with pcDNA3.1 group which cells treated with 100 μmol/L TMZ. ^##^ *P* < 0.01 compared with si‐NC group which cells treated with PBS. ^aa^ *P* < 0.01 compared with si‐NC group which cells treated with 100 μmol/L TMZ. Data was presented as Mean ± SD (standard deviation) from triple experiments

**Figure 5:**
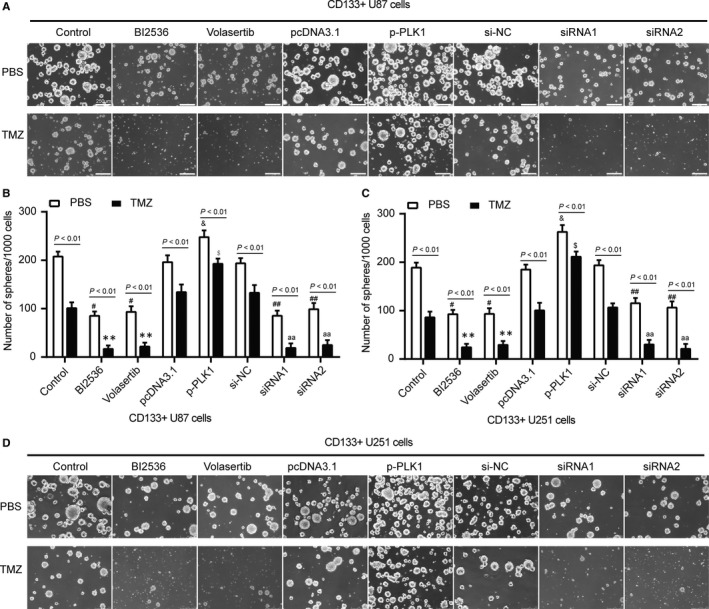
The Influence of TMZ on Sphere Formation of CD133^+^ U87 and CD133^+^ U251 Stem Cells was Investigated. A, During the sphere formation process, PBS or 100 μmol/L TMZ was added into the culture medium. After 8 d, photographs were taken to compare the sphere formation ability of CD133^+^ U87 stem cells. B, After centrifugation, sphere number was calculated. TMZ significantly reduced the sphere number in U87 stem cells with low PLK1 protein expression. C, After centrifugation, sphere number was calculated of CD133^+^ U251 stem cells. D, Sphere formation ability of CD133^+^ U251 stem cells after treated with PBS or 100 μmol/L TMZ for 8 d. Data were presented as Mean ± SD (standard deviation) from triple experiments. ^#^ *P* < 0.05 compared with control group which cells treated with PBS. **P < 0.01 compared with control group which cells treated with 100 μmol/L TMZ. ^&^ *P* < 0.05 compared with pcDNA3.1 group which cells treated with PBS. ^$^ *P* < 0.05 compared with pcDNA3.1 group which cells treated with 100 μmol/L TMZ. ^##^ *P* < 0.01 compared with si‐NC group which cells treated with PBS. ^aa^ *P* < 0.01 compared with si‐NC group which cells treated with 100 μmol/L TMZ

To further verify the effect of *PLK1* on tolerance in glioma stem cells, we inhibited or increased the expression of *PLK1* protein in CD133^+^ U87 stem cells and CD133^+^ U251 stem cells. PLK1 inhibitor BI2536 and Volasertib had no significant impact on PLK1 mRNA expression. PcDNA3.1‐*PLK1* significantly increased *PLK1* mRNA expression, while *PLK1* siRNA1 and siRNA2 significantly inhibited *PLK1* mRNA expression (Figure 4A,B, *P* < 0.01). The results of Western blot showed that BI2536 and Volasertib significantly inhibited the protein level of PLK1. *PLK1* siRNA1 and siRNA2 reduced the expression of PLK1 protein, while pcDNA3.1‐PLK1 raised the expression of PLK1 protein in CD133^+^ U87 stem cells and CD133^+^ U251 stem cells (Figure 4C,D, *P* < 0.01). These nine groups of cells were further treated with PBS and 100 μmol/L TMZ for 48 hours. It was found that TMZ had a greater inhibitory effect on CD133^+^ U87 stem cells and CD133^+^ U251 stem cells in BI2536, Volasertib, *PLK1* siRNA1 and siRNA2 groups, but overexpression of *PLK1* offset TMZ inhibition of CD133^+^ U87 stem cells (Figure 4E,F, *P* < 0.05). Furthermore, downexpression of PLK1 protein by BI2536, Volasertib, *PLK1* siRNA1 and siRNA2 significantly enhanced G2/M arrest in CD133^+^ U87 stem cells and CD133^+^ U251 stem cells after treated with 100 μmol/L TMZ compared with PBS group (Figure 4G,H, *P* < 0.05). Sphere‐forming assays were carried out to explore influence of TMZ on sphere formation of CD133^+^ U87 stem cells and CD133^+^ U251 stem cells. Downexpression of PLK1 significantly decreased the number of CD133^+^ U87 stem cells and CD133^+^ U251 stem cells, enhanced sensibility of CD133^+^ U87 stem cells and CD133^+^ U251 stem cells to TMZ (Figure 5, *P* < 0.05).

### 3.4 PLK1 inhibitor displayed synergistic activity with TMZ to inhibit CD133^+^ U87 stem cells growth in vivo

**Figure 6:**
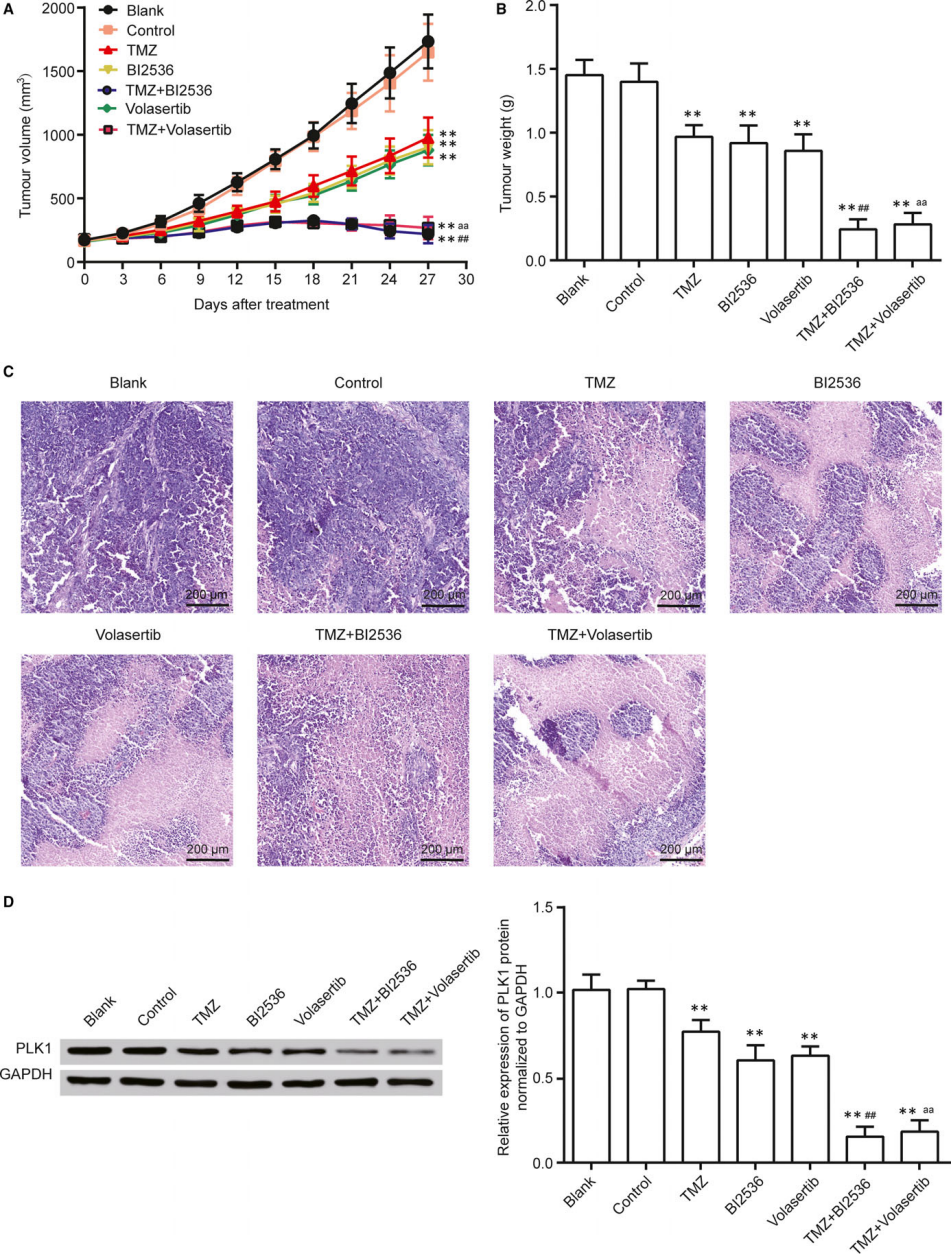
Inhibition of PLK1 Enhanced Sensitivity to TMZ of CD133^+^ U87 Stem Cells In Vivo. Subcutaneous tumours generated from CD133^+^ U87 stem cells were allowed to reach a volume of 150‐200 mm^3^ and were treated with TMZ (50 mg/kg, intraperitoneal injection), BI2536 (40 mg/kg, intravenous injection), Volasertib (15 mg/kg, intravenous injection) or a combination of TMZ and BI2536, TMZ and Volasertib A, Tumour volumes in treatment groups. B, Tumour weights at 27 d after treatment. C, Representative HE staining of different groups was shown. D, The total PLK1 protein expression in tumour tissues was determined by Western blot. **P < 0.01 compared with Blank or control groups. ^##^ *P* < 0.01 compared with BI2536 or TMZ groups. ^aa^ *P* < 0.01 compared with Volasertib or TMZ groups

We also examined whether the combination of PLK1 inhibitor and TMZ displays synergistically anti‐glioma effects in vivo. PLK1 inhibitor (BI2536 and Volasertib) and TMZ observably inhibited CD133^+^ U87 stem cells growth in vivo compared with control or Blank groups. Strikingly, combinatorial treatment with both drugs (TMZ plus BI2536 or TMZ plus Volasertib) resulted in synergistically reduced tumour growth rates (Figure 6A, *P* < 0.01). Tumour weights at 27 days after treatment in BI2536, Volasertib and TMZ groups were markedly reduced in comparison with control or Blank groups. TMZ plus BI2536 or TMZ plus Volasertib treatment lead to stronger reduction compared with BI2536, Volasertib or TMZ treated solely (Figure 6B, *P* < 0.01). There were a large number of necrotic cells, which have undergone karorrhexis (fragmentation) and karyolysis (dissolution) forming a uniform pink area, in the TMZ, BI2536, Volasertib, TMZ plus BI2536 or TMZ plus Volasertib groups. In particular, the pink areas in TMZ plus BI2536 or TMZ plus Volasertib groups were larger than TMZ, BI2536 and Volasertib groups, indicating a synergistic effect of TMZ and PLK1 inhibitors (Figure 6C). Western blot results suggested that PLK1 protein was notably down‐regulated by TMZ, BI2536 and Volasertib treatment and was lower in TMZ plus BI2536 and TMZ plus Volasertib treatment (Figure 6D, *P* < 0.01). These results indicated that inhibition of PLK1 by BI2536 or Volasertib enhanced sensitivity to TMZ of CD133^+^ U87 stem cells in vivo.

## 4 DISCUSSION

This study uncovered the inhibitory effect of TMZ on cell viability and cell cycle of glioma cells and its stem cells on dose‐ and time‐dependent manners. Compared with U87 cells or U251 cells, the stem cells showed greater TMZ resistance. After treatment with TMZ, the expression of *PLK1* protein increased in U87 cells and U87 stem cells. Additionally, a series of experiments showed the synergistic inhibition effect of *PLK1* inhibitor with TMZ on glioblastoma stem‐like cells growth in vitro and *vivo*.

Although immunotherapy with TMZ is widely applied for glioma treatment, the increasing development of drug resistance has become one of the main causes of treatment failure.^15^ A number of papers pointed out that tumour relapse could be because of a restrict population of cells, endowed with tumour initiating potential, which are commonly referred to as glioma stem‐like cells.^16^ Many publications ever reported their researches on the drug resistance of glioma stem cells to TMZ. Alimonte et al elucidated that TMZ caused a dose‐dependent reduction of glioblastoma stem cells survival.^17^ In our study, two types of cells were treated with TMZ to examine whether glioma stem cells are equipped with stronger capability for drug resistance than glioma cells. The results showed that TMZ effectively attenuated the duplication and cell cycle of glioma cells and stem cells in a dose‐dependent manner and had a greater effect on U87 cells and U251 cells, which revealed the presence of different resistant mechanism in glioma cells and glioma stem cells. Consistently, the suppressive effect of TMZ on TJ905 glioma cells was more significant than its effect on TJ905 stem cells.^18^ And Beier et al also discovered that glioblastoma stem cell had stronger resistance to TMZ compared with glioblastoma cells.^4^

Relevant studies have revealed the *PLK1* was involved in the mechanism of cancer growth. For instance, *PLK1* was elevated in glioblastoma multiforme cells and its inhibition suppressed cell growth and induced cell death.^19^ In medulloblastoma, down‐regulation of *PLK1* impaired tumour sphere formation of medulloblastoma cells and induced cell apoptosis.^13^ Additionally, Koncar et al demonstrated that *PLK1* inhibition enhanced TMZ efficacy in IDH1 mutant gliomas.^14^ Additional modalities of TMZ‐resistant such as *PLK1* expression further complicate the mechanism of glioma drug resistance. Hence, we employed experiments to examine the interaction of *PLK1* and TMZ in TMZ‐resistant glioma cells, and found that *PLK1* expression was impeded with the increase in TMZ concentration. Furthermore, *PLK1* inhibitor or knockdown of *PLK1* facilitated the inhibitory effect of TMZ on cell viability and cell cycle.

Although we have elucidated the links between *PLK1* expression and TMZ efficacy on U87 and U251 stem cells, the current study also has some unavoidable limitations. For example, other molecules involved in drug‐resistant glioma stem cells are worthy to be studied.

In summary, our study proposed the synergistic inhibition effect of *PLK1* inhibitor with TMZ on glioblastoma stem‐like cells and suggested the critical role of *PLK1* in glioma stem cells progression, providing new treatment strategies for gliomas.

## CONFLICT OF INTEREST

The authors confirm that there are no conflicts of interest.
